# In Situ Sintering of CdSe/CdS Nanocrystals under Electron Beam Irradiation

**DOI:** 10.3390/nano13243082

**Published:** 2023-12-05

**Authors:** Luping Tang, Chun Zhang, Chen Liao, Yiwei Liu, Yonghao Cheng

**Affiliations:** 1 College of Mechanical and Electrical Engineering, Nanjing Forestry University, Nanjing 210037, China; 2 SEU-FEI Nano-Pico Center, Key Lab of MEMS of Ministry of Education, Southeast University, Nanjing 210096, China; 3 College of Electronic and Optical Engineering & College of Flexible Electronics (Future Technology), Nanjing University of Posts and Telecommunications, Nanjing 210023, China; 4 College of Electrical Engineering, Yanshan University, Qinhuangdao 066004, China

**Keywords:** electron beam irradiation, CdSe/CdS nanocrystals, sintering, surface atom diffusion, transmission electron microscopy

## Abstract

Colloidal semiconductor nanocrystals have attracted widespread attention due to their tremendous electrical and optical properties. Nanoparticles exhibit a strong tendency to aggregate and sinter in a short period of time during processing or use due to their large surface area-to-volume ratio, which may lead to significant changes in their required performance. Therefore, it is of great significance to conduct in-depth research on the sintering process and mechanism of nanoparticles to maintain their stability. Here, the sintering process of CdSe/CdS core/shell nanocrystals under continuous electron beam irradiation was studied using in situ transmission electron microscopy (TEM). In the early stages of sintering, CdSe/CdS nanocrystals approached each other at a distance of approximately 1–2 nm. As the exposure time to the electron beam increased, the movement of surface atoms on the nanocrystals led to contact between them. Subsequently, the atoms on the contact surfaces underwent rapid motion, resulting in the rapid formation of the neck between the particles. The neck formation between adjacent particles provides strong evidence of a sintering mechanism dominated by surface atom diffusion rather than Ostwald ripening. Further research in this area could lead to the development of improved methods to prevent sintering and enhance the stability of nanocrystals, ultimately contributing to the advancement of nanomaterial-based devices and materials with long-lasting performance.

## 1. Introduction

Colloidal nanocrystals, especially CdSe/CdS core/shell heterostructures, have been demonstrated as a versatile class of nanomaterials with potential applications in lasers [1,2], light-emitting diodes [3,4], bio-labeling [5], and single-photon sources [6]. However, in many of these applications, maintaining the thermal stability of nanocrystals is crucial [7,8,9]. Due to their high surface area-to-volume ratio, nanocrystals tend to undergo strong aggregation and sintering tendencies in a short time during processing or usage, which can lead to significant changes in their desired performance, subsequently affecting their behavior and properties [10]. Therefore, the sintering process of nanocrystals is an interesting and essential topic for assessing the stability of colloidal nanocrystals. The relevant knowledge can help us develop methods to prevent sintering, thereby enhancing the stability of nanocrystals [11,12].

So far, various techniques, including thermal [13], electrical [14], laser [15], microwave [16], plasma [17], and electron beam [18,19,20] methods, have been applied to explore the sintering of metal nanocrystals. For example, Liu et al. demonstrated that under high-energy electron beam irradiation, Au and Ag nanocrystals come into close contact, forming physical contact. This is because energy transfer from the electron beam to the nanocrystals can induce the diffusion and sintering of Au and Ag nanocrystals [18]. Cichocka et al. observed Pt nanocrystal sintering within thin-walled carbon nanotubes using transmission electron microscopy with aberration correction under electron beam irradiation at an accelerating voltage of 80 kV [21]. TEM heating has been used to study the effect of carbon surface coatings on the sintering of silver nanocrystals, indicating that carbon surface coatings can significantly inhibit the sintering of silver nanocrystals [22]. Many efforts have been made to clarify the energy sources and growth mechanisms for the aggregation of metal nanocrystals. From a thermodynamic perspective, a substantial body of research suggests that a reduction in surface energy is the primary thermodynamic driving force for the aggregation of metal nanocrystals [23,24]. From a kinetic perspective, researchers have used advanced in situ characterization methods to deeply discuss the aggregation dynamics of metal nanocrystals during the aggregation process [25,26,27]. However, these works have mainly focused on the sintering process of metal nanoparticles, and the sintering processes and mechanisms of semiconductor nanoparticles are not yet clear.

Moreover, the self-assembly of nanoparticles in a controlled manner indicates the probability of employing nanoparticles as building blocks to create nanoscale structures and new device components. Electron beam irradiation has been demonstrated to be an important way to modify the assembly of nanoparticles. For example, electron beam writing has been used to create patterns and lines in alkylthiol-passivated gold nanoparticle films, resulting in the formation of carbon networks in which gold cores are distributed [28]. Wires can be acquired by the sintering of metal nanoparticles assembled on a linear template, in which the organic ligands are removed through electron beam irradiation [29]. The electron beam sintering method uses electron beam irradiation to sinter samples, facilitating single crystal sintering. Its advantage is that it can accurately process the sample and has a high energy density. In addition, the study of electron irradiation effects based on transmission electron microscopy is beneficial for exploring the structural stability and evolution of materials under electron irradiation at the atomic scale, as well as deepening the understanding of the electron irradiation process and providing a theoretical and experimental basis for high-precision and controllable processing of nanostructures.

This paper conducted in situ TEM heating and electron beam irradiation experiments, allowing us to observe the sintering process of semiconductor nanocrystals in real-time. By growing approximately 16 layers of CdS shell on CdSe seeds through successive ionic layer adsorption and reaction (SILAR), CdSe/CdS nanocrystals with a nominal size of 15 nm were obtained. Electron beam-induced nanoparticle movement controlled the convergence and sintering of particles. Specifically, the formation of a neck between adjacent CdSe/CdS nanocrystals demonstrates a mechanism driven by surface atom diffusion in the initial sintering stage rather than Ostwald ripening or diffusion collision of nanocrystals. In addition, the stability of the dimer produced by sintering is also further investigated. This study also suggests that when TEM is used for characterizing nanomaterials, high electron beam intensity and long exposure times should be avoided, especially for materials with low surface binding energy.

## 2. Materials and Methods

### 2.1. Preparation of CdSe/CdS Nanocrystals

Approximately 16 monolayers of CdS shells were grown on CdSe grains through SILAR on the CdSe nanocrystal cores, resulting in solid CdSe/CdS core/shell structure nanocrystals [30,31]. The CdSe nanocrystal cores were obtained in a conventional way, with the additional procedural details available in reference [30]. The CdSe/CdS core/shell nanocrystals were synthesized employing the SILAR method. The cadmium precursor solution (0.1 M) was acquired by combining CdO (0.385 g), oleic acid (6.778 g), and trioctylphosphine oxide (17.64 g), resulting in a clear solution under an argon atmosphere at 250 °C. Simultaneously, the sulfur precursor solution (0.1 mol/L) was formulated by dissolving sulfur (0.096 g) in trioctylphosphine oxide (23.67 g) under an argon atmosphere at 100 °C. The quantity of sulfur or cadmium precursors required for each monolayer was determined based on the volume increment of each shell and the nanoparticle concentration of the nanocrystals. The CdSe nanocrystals were combined with octadecane (30 g) and oleylamine (10 mL) in a three-necked flask. Following the hexane removal at 60 °C under vacuum, the mixture underwent heating to 240 °C under an argon flow, initiating the growth of 1–5 monolayers of the shell. The CdSe/CdS core/shell nanocrystals were formed through the sequential addition of the cadmium precursor and the sulfur precursor at intervals of 10 min. For the growth of 6–15 monolayer shells, the temperature was elevated to 280 °C, and the interval between each addition was increased to 30 min. Subsequently, the obtained nanocrystals underwent washing and purification through precipitation two to three times with ethanol, followed by redispersion in hexane.

### 2.2. In Situ TEM Heating and Characterization

A heating stage utilizing a micro-electro-mechanical system (MEMS) device was used as both a heating element and a sample support grid [32]. The support grid had an electrical feedthrough connected to an external power source. The synthesized nanocrystals were first sonicated for ten minutes in an ultrasonic bath to reduce particle aggregation. Then, 5 μL of a diluted CdSe/CdS nanocrystals solution was deposited onto an Adurot heating chip using a micro-pipette, which was then mounted on a Protochips in situ heating holder and exposed to the electron beam in a FEI Titan 80–300 TEM operated at 300 kV and room temperature.

## 3. Results and Discussion

**Figure 1:**
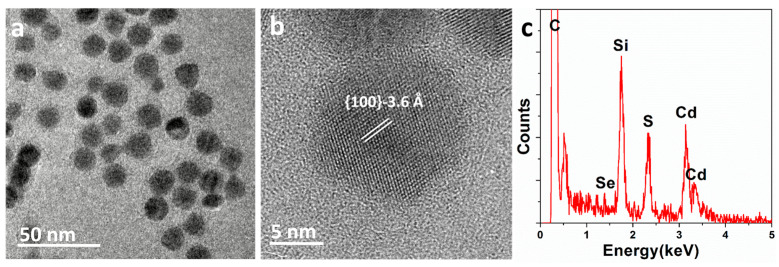
(**a**,**b**) Transmission electron microscopy (TEM) image and high-resolution TEM (HRTEM) image of the CdSe/CdS nanocrystals; (**c**) energy-dispersive X-ray spectroscopy (EDX) spectrum of the CdSe/CdS nanocrystals, revealing the high chemical purity.

The TEM images of the CdSe/CdS core/shell nanocrystals synthesized using the SILAR method are shown in Figure 1a and 1b. These images indicate a fairly uniform size distribution of nanocrystals, with an average particle size of approximately 15.4 nm. High-resolution TEM images (Figure 1b) reveal good crystallinity, with lattice fringes extending throughout the quantum dots. The interplanar spacing observed in the TEM images is 3.6 Å, consistent with the interplanar spacing of the CdS (100) surface in the zinc blende phase. Moreover, energy-dispersive X-ray spectroscopy (EDX) analysis (Figure 1c) indicates the high chemical purity of these samples. Strong signals for cadmium and sulfur are detected in the spectrum, with weak signals for selenium, while peaks from carbon and silicon from the TEM grid are also detected. The peak for oxygen is negligible. Initially, several regions of interest were selected and labeled on the TEM stage. Electron beam irradiation caused the surface-active agent molecules on the CdSe/CdS nanocrystal surfaces and carbonaceous molecules within the TEM chamber to become carbonized in the imaging area, subsequently forming a carbon shell on the CdSe/CdS nanocrystal surfaces.

**Figure 2:**
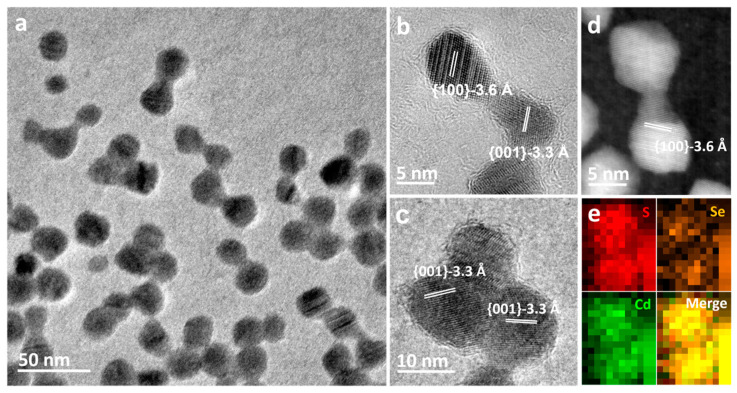
(**a**) TEM image of CdSe/CdS nanocrystals taken after electron beam irradiation for 15 min at room temperature. (**b**,**c**) HRTEM images of sintering nanoparticles involving only two and three adjacent nanoparticles. (**d**) Representative high-angle annular dark field-scanning TEM (HAADF-STEM) images of a dimer displaying lattice fringes with spacing corresponding to CdSeS ({100}-3.6 Å). (**e**) Se, Cd, and S elemental maps acquired from EDX mapping showing that the elements are nearly uniformly distributed in the dimer.

Figure 2 displays bright-field electron micrographs of a small area of nanocrystals after electron beam irradiation at 300 kV. Further electron beam irradiation led to the formation of single, fully crystalline new particles, along with a significant contraction in the sizes of aggregates formed due to coalescence, as shown in Figure 2a. Sintering was observed to occur only between particles with narrow gaps of approximately 1–2 nm. Even after extended electron beam exposure, sintering remained confined to a small region, typically involving only two or three adjacent nanocrystals. The increased spacing between sintered particles over time hindered continuous sintering, confirming the limited mobility of particles. The 3.6 Å and 3.3 Å spacing fringes in Figure 2b,c correspond to the {100} and {001} lattice fringes of CdSeS, respectively. High-resolution HAADF-STEM image of the dimer in Figure 2d confirms the lattice fringes of CdSeS{100} planes are clearly visible. The EDX image of the dimer in Figure 2e shows a uniform distribution of all three elements, indicating that the dimeric structure of CdSe/CdS nanocrystals eventually converts to stoichiometric CdSeS.

**Figure 3:**
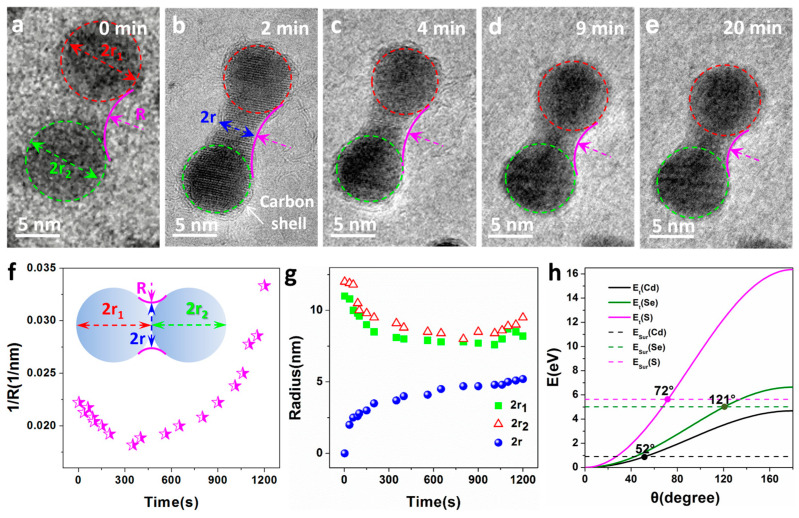
(**a**–**e**) A sequence of in situ TEM heating images displaying sintering of two CdSe/CdS nanocrystals under electron beam irradiation at room temperature. The arrow in the second frame shows a carbon shell presented on the surface of the dimer. (**f**,**g**) Variation of parameters, including neck curvature (1/R), radius of individual particles ($r_{1}$ and $r_{2}$) and the neck (r) as a function of electron beam irradiation time, a model established for the newly formed dimer with some parameters defined. The illustration shows the model established for the newly formed dimer, which defines some parameters. (**h**) Transfer energy ($E_{t}$) of Cd (black), Se (green), and S (pink) from 300 keV electrons as a function of the scattering angle θ. Horizontal dashed lines show the surface binding energy of Cd (black), Se (green), and S (pink).

The sintering process of CdSe/CdS nanocrystals under continuous electron beam irradiation was investigated by in situ TEM. The sequence of images in Figure 3a–e shows two CdSe/CdS nanocrystals of similar size (approximately 15 nm) after different durations of exposure to the electron beam at room temperature. Initially, the two nanocrystals were in separate positions at a certain distance, as shown in Figure 3a. It is worth noting that a carbon shell is clearly present on the surface of the nanocrystals, as shown by the red arrows in Figure 3b. CdSe/CdS nanocrystals obtained using chemical methods often adsorb a layer of surface-active agent molecules on their surfaces, which become carbonized under electron beam irradiation. Simultaneously, carbonaceous molecules on the support membrane of the electron chip/grid can diffuse into the imaging area and also carbonize to facilitate the growth of the carbon shell on the surfaces. Initially, there was no significant translational motion between the two nanocrystals in the early irradiation stage, but once they came into contact, a neck formed rapidly over the next 2 min, with the neck growth rate gradually slowing down over the next 20 min.

To better describe the sintering process and understand its growth mechanism, a dimer model was established (inset of Figure 3f), and several parameters were defined. The geometric definitions of $r_{1}$, $r_{2}$, R, and r are as shown in Figure 3f. Parameters related to the curvature of the neck as a function of time (1/R), the radii of the CdSe/CdS nanocrystals ($r_{1}$ and $r_{2}$), and the neck region radius (r) are illustrated in Figure 3f,g. These measurable parameters shown in Figure 3f,g were extracted from a series of TEM micrographs taken at different time intervals. In order to better describe the changes in 1/R, $r_{1}$, and $r_{2}$ during the sintering process, the nanoparticles in Figure 3a–e were approximated as spheres and marked with red and green dashed circles, as shown in Figure 3. The changes in 1/R, $r_{1}$, and $r_{2}$ with sintering time can be clearly seen from the figure. In the early stages of sintering, CdSe/CdS nanocrystals approached each other at a distance of approximately 1–2 nm. As the exposure time to the electron beam increased, the movement of surface atoms on the nanocrystals led to contact between them. Subsequently, the atoms on the contact surfaces underwent rapid motion, resulting in the rapid formation of the neck between the particles. This caused the parameter  1/R to decrease rapidly with increasing exposure time to the electron beam, as seen in Figure 3a–c, and also led to a decrease in the radii $r_{1}$ and $r_{2}$. With a further increase in exposure time, the surface atoms at the ends of the two nanocrystals in contact, far from the neck, gradually moved towards the neck, promoting further neck growth. This led to a gradual increase in 1/R with increasing exposure time, while $r_{1}$ and $r_{2}$ slightly increased, as shown in Figure 3c–e. Regarding r, in the early stages of sintering, particularly within the initial 2 min (see Figure 3a,b), the growth rate of the neck diameter r exhibited a rapid growth relative to the exposure time, followed by linear growth. This behavior was primarily driven by the highly active surface associated with the large neck curvature in the dimer. In addition, regarding the total volume change before and after sintering, only approximate calculations can be made due to the obvious necking formed by the sintering of nanoparticles. By treating CdSe/CdS nanocrystals as approximate spheres for calculation, it was found that there was no significant change in the total volume before and after sintering (including the neck).

Sintering of bare nanoparticles on a support is typically modeled by “Ostwald ripening”, where individual atoms leave one nanoparticle, diffuse on the support, and attach to another nanoparticle [33]. The growth of larger particles comes at the expense of smaller particles. However, when interactions between nanoparticles and the support are weak, the diffusion of entire nanoparticles becomes possible, leading to sintering by particle migration and collision with other particles [18,34]. In situ observations confirm that once in contact, a pair or a group of nanoparticles coalesce to reduce their surface energy. As shown in Figure 3, the neck formation between adjacent particles provides strong evidence of a sintering mechanism dominated by surface atom diffusion rather than Ostwald ripening. The migration of surface atoms plays a crucial role in this sintering process, especially for small particles with a large ratio of surface atoms to volume atoms [35]. During electron beam excitation, the electron beam causes heating and sputtering, which are the two major effects [36]. Despite the fact that electron beam illumination may generate heat, the temperature only increases by a few degrees, and the thermal effect is always minimal [37]. Therefore, electron beam sputtering caused by the energy transfer of deflected electrons plays an important role in driving the sintering of nanoparticles. To estimate the electron beam sputtering effect, a model based on electron scattering was applied to determine the energy $E_{t}$ transmitted through electron atom collisions, which is obtained by [37,38]

(1) $$E_{t}=\frac{2E_{0}\left(E_{0}+2mc^{2}\right)}{Mc^{2}}\cdot {\left(\sin\frac{\theta }{2}\right)}^{2}$$

where m, M, θ, $E_{0}$, and c are the values of electron mass, atomic mass, scattering angle, electron energy, and speed of light in vacuum, respectively. Substituting the corresponding parameters for Se, S, and Cd, the relationship of Et as a function of θ is calculated and plotted in Figure 3h. The transmitted energy increases monotonically with the increase in the scattering angle. Although most electrons scatter at small angles with negligible transfer energy (<1 eV), the backscattering of electrons (θ > 90°, even θ = 180° is unavoidable, which leads to significant energy transfer and has a significant impact on nanoparticles made of elements, especially low-atomic-weight elements. When the transmitted energy ($E_{t}$) is greater than the surface binding energy, atoms on the surface of nanoparticles can diffuse when irradiated with an electron beam [18]. The surface atoms on CdSe/CdS nanocrystals can diffuse under electron beam irradiation, as the transfer energy $E_{t}$ of Cd, S, and Se, can exceed their surface atomic binding energies (0.90, 5.63, and 5.01 eV, respectively); namely, the electron deflection angles are greater than 149°, 65°, and 107°, respectively (at the intersection of the horizontal solid and dashed lines in Figure 3h).

**Figure 4:**
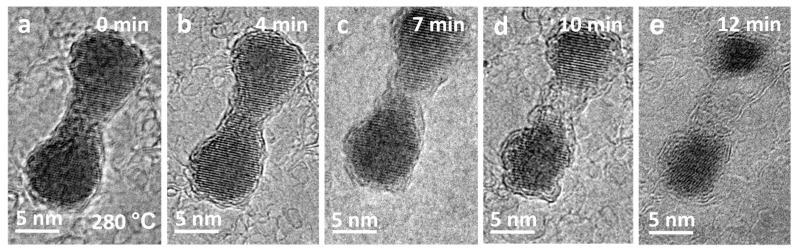
(**a**–**e**) HTEM image sequence displaying the sublimated progression of newly formed dimer at 280 °C.

Electron beam irradiation of CdSe/CdS nanocrystals can successfully produce coupled colloidal nanostructures through sintering. Recently, it has already been demonstrated that coupled colloidal quantum dots could show a new type of color-switchable red–green display pixel, which could potentially simplify the classic RGB display production or as an electric field-induced color-tuned single-photon source [39]. Since the stability of such coupled colloidal quantum dots is very important when applied in applications [40,41], it is necessary to obtain detailed information on the thermal and temporal behavior of these structures. Figure 4 presents a series of HRTEM images that depict the continuous stages of phase transition and morphological changes of the CdSe/CdS dimer formed after sintering at higher temperatures. During subsequent annealing at 280 °C, as shown in Figure 4a–e, Cd, Se, and S atoms evaporated, causing sublimation of the crystalline CdSeS dimer, resulting in the dimer’s contraction, ultimately causing the dimer to nearly disappear (Figure 4e). Lattice fringes of the dimer remained present throughout the sublimation process, indicating that the dimer consistently maintained its crystalline state without undergoing a crystal-to-liquid transition, i.e., melting.

## 4. Conclusions

Nanocrystals, due to their large specific surface area, are prone to agglomeration or sintering in a short time during use and processing, resulting in significant changes in their performance. This article conducted in situ electron microscopy studies on the sintering of CdSe/CdS nanocrystals under electron beam irradiation. In situ observations confirm that there was no significant translational motion between the two nanocrystals in the early irradiation stage, but once they came into contact, a neck formed rapidly. Sintering of bare nanoparticles on a support is typically modeled by “Ostwald ripening”, where individual atoms leave one nanoparticle, diffuse on the support, and attach to another nanoparticle. The growth of larger particles comes at the expense of smaller particles. While in this sintering mechanism, CdSe/CdS nanocrystals are either close enough to each other or able to move freely, leading to collisions with other particles, resulting in coalescence. The neck formation between adjacent particles is strong evidence of this mechanism. The migration of surface atoms plays a crucial role in this sintering process, especially for small particles with a large ratio of surface atoms to volume atoms. Moreover, the newly formed CdSe/CdS dimers would sublimate at 280 °C. Understanding the sintering process of colloidal semiconductor nanocrystals is crucial for the long-term performance of micro-nanodevices and materials based on nanocrystals.

## Data Availability

Data are contained within the article.
